# The impact of persistent precarity on patients’ capacity to manage their treatment burden: A comparative qualitative study between urban and rural patients with multimorbidity in South Africa

**DOI:** 10.3389/fmed.2023.1061190

**Published:** 2023-03-30

**Authors:** Myrna van Pinxteren, Nonzuzo Mbokazi, Katherine Murphy, Frances S. Mair, Carl May, Naomi Levitt

**Affiliations:** ^1^Chronic Disease Initiative for Africa, Department of Medicine, University of Cape Town, Cape Town, South Africa; ^2^School of Health and Well-Being, College of Medical, Veterinary and Life Sciences, University of Glasgow, Glasgow, United Kingdom; ^3^Department of Health Services Research and Policy, London School of Hygiene and Tropical Medicine, London, United Kingdom; ^4^NIHR North Thames Applied Research Collaboration, London, United Kingdom

**Keywords:** multimorbidity (co-morbidity), treatment burden, qualitative research and analysis, low-income context, precarity

## Abstract

**Background:**

People living with multimorbidity in low-and middle-income countries (LMICs) experience a high workload trying to meet the demands of self-management. In an unequal society like South Africa, many people face continuous economic uncertainty, which can impact on their capacity to manage their illnesses and lead to poor health outcomes. Using precariousness – the real and perceived impact of uncertainty – as a lens, this paper aims to identify, characterise, and understand the workload and capacity associated with self-management amongst people with multimorbidity living in precarious circumstances in urban and rural South Africa.

**Methods:**

We conducted qualitative semi-structured interviews with 30 patients with HIV and co-morbidities between February and April 2021. Patients were attending public clinics in Cape Town (Western Cape) and Bulungula (Eastern Cape). Interviews were transcribed and data analysed using qualitative framework analysis. Burden of Treatment Theory (BoTT) and the Cumulative Complexity Model (CuCoM) were used as theoretical lenses through which to conceptualise the data.

**Results:**

People with multimorbidity in rural and urban South Africa experienced multi-faceted precariousness, including financial and housing insecurity, dangerous living circumstances and exposure to violence. Women felt unsafe in their communities and sometimes their homes, whilst men struggled with substance use and a lack of social support. Older patients relied on small income grants often shared with others, whilst younger patients struggled to find stable employment and combine self-management with family responsibilities. Precariousness impacted access to health services and information and peoples’ ability to buy healthy foods and out-of-pocket medication, thus increasing their treatment burden and reducing their capacity.

**Conclusion:**

This study highlights that precariousness reduces the capacity and increases treatment burden for patients with multimorbidity in low-income settings in South Africa. Precariousness is both accumulative and cyclic, as financial insecurity impacts every aspect of peoples’ daily lives. Findings emphasise that current models examining treatment burden need to be adapted to accommodate patients’ experiences in low-income settings and address cumulative precariousness. Understanding treatment burden and capacity for patients in LMICs is a crucial first step to redesign health systems which aim to improve self-management and offer comprehensive person-centred care.

## Background

Precariousness, the real and perceived impact of uncertainty, is a concept originally developed to determine the effects of economic insecurity and absence of state support on people’s lives (1). It has since been extended to include the impact on people living under the threat of natural disasters and experiencing a lack of social and economic rights (1). Faced by continuous hardship, people who are “living on the edge” are more at risk of poverty, starvation, displacement, and exposure to violence without protection (2–4). Precariousness profoundly impacts people’s health and well-being by reducing people’s access to health care, health information and support networks (5–8). However, the impact of precariousness on patients with multimorbidity in low-middle countries (LMICs) is underexplored.

South Africa is a country with significant social and economic inequalities, scoring 63 on the Gini Coefficient index, amongst the highest in the world (9). These inequalities are caused by colonialization and the legacy of racial discrimination during apartheid, which deprived people of educational opportunities, jobs, adequate housing, and access to health care based on their racial classification (10–13). Almost 30 years post-apartheid, 34.5% of South Africans are unemployed, one in five South Africans rely on social welfare grants from the state (10, 14) and almost 20% of South African households have inadequate access to sufficient safe, and nutritious food (15). An estimated 25% of South Africans live in a township or slum setting and 14% of the population resides in informal dwellings, commonly called shacks (16). These social disparities, result in an increased burden of disease and poor health outcomes (10, 17).

With more than 8 million people receiving antiretroviral therapy (ART), South Africa has the highest number of people living with HIV (PLWH) in the world, whilst simultaneously experiencing a rising burden of non-communicable diseases (NCDs), most commonly diabetes, cardio-vascular diseases, hypertension and mental illness (18–21). These colliding epidemics are giving rise to patterns of multimorbidity, defined as two or more chronic conditions, disproportionately affecting socio-economically disadvantaged populations. The rising prevalence in multimorbidity has societal consequences, impacting the livelihood and futures of working populations and families (22). In the past 15 years, steep increases in NCDs have been reported in low-income groups, who also have the highest burden of HIV (23–25). Currently, of those seeking care in South Africa, the estimated prevalence of multimorbidity is between 22.6 and 48.4% (21, 26–28). More than 80% of South Africans use free public, state funded health services which are fragmented and not equipped to provide care for the long-term and complex treatment of multimorbidity patients (22, 29). Currently, no integrated care is offered for people living with HIV/NCD multimorbidity, and administration and consultation are taking place in separate clinics on separate days (30, 31). However, antiretroviral (ART) adherence clubs and differentiated HIV service delivery are offered free of charge to reduce waiting times and overcrowding in clinics (30, 32, 33). Availability of, access to and the quality of health services also differ between provinces and between urban and rural areas, underserving rural low-income households (34). Longstanding health system weaknesses have been exacerbated during the COVID-19 pandemic, as clinics are dealing with stock-outs of medication, interrupted services and staff shortages (35).

Responding to the rising burden of chronic communicable and NCDs in South Africa and increased prevalence of multimorbidity, the National Department of Health (NDoH) has developed initiatives to improve the integration of services at a primary care level, such as the Integrated Chronic Disease Management model (ICDM) (36). The ICDM model, informed by the WHO Innovative Care for Chronic Conditions Framework (ICCC), aims to streamline health care programmes on different levels: improve patients interactions on the micro-level, increase health organisation and community engagement on the meso-level, and restructure policies at the macro-level (37). It emphasises that optimal outcomes are achieved when there is both a partnership between patients, communities and health services and patients are empowered to take responsibility for their health. The ICDM model is currently being rolled out in 42 clinics in three of the nine South African provinces. Early evaluation studies have revealed a concern that patients with complex chronic conditions are expected to take on more responsibility for self-management, without receiving ongoing support delivered by health services (37, 38).

To further investigate the impact of multimorbidity in patients’ lives, this paper explores the treatment burden experienced by people living with multimorbidity in South Africa, building on two theoretical models, the Burden of Treatment Theory (BoTT) and Cumulative Complexity Model (CuCoM) (39, 40). CuCoM explores the interaction between individual patients’ workload and their capacity to manage this workload, whilst BoTT examines the role of the patient and their supporters in managing, caring, and supporting the work of being a patient and analyses how patient workload is distributed through and within social networks (39–41). These models have been developed and tested in high income countries and research on treatment burden in LMICs is scarce, but the few qualitative studies done in Sub-Saharan Africa report that patients with multimorbidity face several challenges when navigating their workload, due to inconsistent access to and poor quality of health care, insufficient health knowledge and stigma (30, 42, 43).

Applying both BoTT and CuCoM as a theoretical focus, this paper explores patients’ agency to mobilise social and economic resources to effectively self-manage multimorbidity, in settings of precarity, and aims to understand if and how an absence of these resources can overwhelm patients and increase treatment burden, which in turn can lead to complications and ill-health (39, 44, 45).

## Materials and methods

### Study design

This paper is one of the outputs of a qualitative study “Exploring the treatment burden and capacity for self-care amongst people living with HIV/NCD multimorbidity in South Africa to inform the development of interventions to reduce workload and improve capacity” (EXTRA) (46). We used the method of in-depth, qualitative, individual interviews, which allowed an interpretivist narrative approach where participants were encouraged to share their life-stories and personal experiences of living with multimorbidity (47, 48). We used a semi-structured interview guide with open-ended questions adapted from the Burden of Treatment questionnaire (BTQ), BoTT and concepts from the CuCoM to explore individual patient and carer experiences (39, 40, 49). Questions were focused on the structural, spatial, and systemic factors that impact on patients’ health care engagement and probed the role of social support networks in patients’ self-management and how they coped with treatment burden (45, 50, 51). An extensive description of the methodological considerations is published elsewhere (46).

### Setting

This study was conducted in two communities of low socio-economic status in South Africa, Gugulethu and Bulungula. The urban township of Gugulethu is located 15 km from Cape Town, in the Western Cape province. Gugulethu was established under the Group Area Act in 1953 as a segregated residential area for black Africans (52, 53). It is home for more than 100,000, mostly isiXhosa speaking residents who live in formal housing or shacks, as backyard dwellers. Compared to many other townships, Gugulethu is well-serviced, and schools, shops and clinics are easily accessible. Bulungula is a remote rural area in the coastal region in the Eastern Cape, previously part of a “homeland” designated for isiXhosa speaking black Africans under apartheid and ruled by proxy through traditional chiefs (54). It is one of the poorest and most underdeveloped areas in the country, with most people still living in traditional type housing with poor infrastructure, a shortage of basic amenities and few economic opportunities (55, 56). Health status in the Eastern Cape is poor: an estimated 20% of the population has HIV and hypertension prevalence rates are 49.8% (57). Women in the Eastern Cape also have the highest prevalence of diabetes in the country (18%) (57). In comparison, in the Western Cape, the estimated prevalence is 18% for HIV and 51.6% for hypertension (57). In addition, men in the Western Cape have the highest prevalence (13%) of diabetes in the country (57).

### Data collection

Data was collected between February and April 2021. Participants were recruited from both and urban and rural South African township, allowing us to examine the differences in people’s lived experiences and narratives (58). As experiences also might differ by age and sex, we included both females and males with various ages, socio-economic backgrounds, and education levels, using a purposive sampling method (59). The inclusion criteria were patients older than 18 years, with a confirmed diagnosis of HIV and at least one other chronic non communicable disease who use public health services In Gugulethu, participants were recruited from one community clinic (Gugulethu CHC). Potential participants were approached by a designated fieldworker who explained the study in everyday language and handed out an information sheet. If the person was interested, the fieldworker would collect phone numbers and set up an appointment for the interview (46). In Bulungula, participants and carers came from two villages supported by the Bulungula Incubator (BI), a local non-governmental organisation. Here, participants were approached by community health workers (CHWs) and when expressing interest, the interviews would be arranged. Semi-structured interviews were conducted by two researchers, Nonzuzo Mbokazi (NM) and Myrna van Pinxteren (MvP) in English or isiXhosa, two of the 11 official languages in South Africa. Each interview lasted between 30 and 90 min and was conducted in a COVID-19 compliant space in the clinic (Gugulethu) or in participants’ homes (Bulungula) (60). All COVID-19 regulations were observed, including masking and social distancing (61). Questions focused on respondents’ understanding of their conditions, experiences of the health services and relationships with their support networks. All interviews were audio-recorded and transcribed and translated by NM and MvP to form the data for analysis.

### Data analysis

Informed by the BoTT and CuCoM, we undertook both inductive and deductive qualitative thematic analysis, guided by a framework approach (62–64). Data analysis was a constant interpretative process, consisting of the following steps: familiarisation, development of coding framework, charting, and further mapping and interpretation (65, 66). Included in the familiarisation phase was data management, assigning initial or open codes to each meaning unit in the transcripts to develop an understanding of what the participants are saying, and developing and refining a coding matrix or organisational schema, to then apply across the data set (46). To understand how, and to which extent concepts explored in BoTT and CuCoM were applicable to our context, we outlined the broad categories using the following constructs: (1) control conditions and enacting control, (2) structural factors, (3) patient capacity, (4) quality of life, and (5) impact of COVID-19. These categories derived from the open coding process and helped us organise, summarise, and condense the data. Table 1 outlines the different domains of treatment burden and elaborates on how these domains were experienced by our participants in urban and rural South Africa. Using these broad categories, we then moved on to a deeper description, explanation and interpretation of the data and developed themes and sub-themes. To ensure rigour and trustworthiness of the study findings, we developed full transcripts that were checked by both MvP and NM for quality control. MvP and NM also developed fieldnotes and narrative memos comprised of summaries of data plus their own reflections and emerging interpretations, which were regularly discussed with all authors to further advance analysis (46). Further details on data analysis, reflexivity and positionality can be found in the qualitative methods paper (46).

**Table 1:** Domains of treatment burden and the experiences of participants in Gugulethu and Bulungula.

Domains of treatment burden (BoTT and CuCoM)	Experiences of participants
Control conditions and enacting control: Including: sense-making (coherence), Practical help (skill-set workability), material and cognitive practices (interactional workability), enacting delegating work (collective action), monitoring (reflexive monitoring)	Learnings about conditions and treatments, how participants undertake self-care and what is required by participants to control conditions (including medication, clinic appointment and seeking health information)
Structural factors: Including: Exploitable resources (contextual integration), social capital (informational and material resources), opportunity (constraints agency), and control over services (structures agency)	Impact of socio-economic factors on the workload for participants, including the organisation of and access to healthcare facilities, relationships with HCPs, living conditions, geography, and culture
Patient capacity: Including: Building and retaining relational networks (extends agency), agency (general potential), social skill (securing co-operation) and structural resilience (potential to absorb adversity)	Explored participants’ social networks, their individual capacity to cope with diagnoses and self-management, including individual agency, resilience, spiritual faith, and strategies to overcoming barriers
Quality of life: Including: expressing capacity, functional performance (potential to do the work)	Unpacked the impact of multiple chronic conditions on participants’ physical, emotional, social, and financial wellbeing
Impact of COVID-19: Including: control over services, social capital (informational and material resources), mobilising capacity and opportunity (constraints agency)	Investigated the impact of COVID-19 lockdowns and regulations on participants’ access to care and medication, social networks, and available economic resources

### Ethical aspects

This study strictly followed the guidelines from the Principles of Good Clinical Practice and the Declaration of Helsinki (67). Ethical approval for this study was obtained from the University of Cape Town (HREC 232/2020) and access to clinics was granted by the Western Cape Department of Health. In the Eastern Cape, we received approval from the (BI) to approach participants identified by community health workers (CHWs). Further detail of ethical considerations are published elsewhere (46).

## Findings

We conducted a total of 30 semi-structured interviews with participants living with HIV and co-morbidities, 16 in Gugulethu, Western Cape and 14 in Bulungula, Eastern Cape (Tables 2, 3). We interviewed 9 men (2 in Bulungula, 7 in Gugulethu) and 21 women (9 in Gugulethu, 12 in Bulungula). All participants were South African citizens and spoke isiXhosa as their first language. The mean age was 56 years in Gugulethu and 50 years in Bulungula. Only one patient received private health care, 29 attended public health services. The most common co-morbidity was hypertension.

**Table 2:** Participants characteristics Gugulethu, Cape Town.

Study ID	Sex	Age	Employment	Time HIV+	Co-morbidity	Carer present	Main type of support
PU001	Female	60	Unemployed	15 years	Diabetes/Asthma/Hypertension, Heart Condition	Yes	Cousin
PU002	Female	62	Unemployed	21 years	Diabetes/HIV	Yes	Son
PU003	Female	53	Retired	14 years	Stroke/Arthritis/ Asthma/ Depression/Hypertension	Yes	Daughter
PU004	Female	48	Unemployed	17 years	HIV/Hypertension/cellulitis	Yes	Friend
PU005	Female	63	Retired	25 years	HIV/Hypertension/TB in Hip	Yes	Partner
PU006	Female	61	Unemployed	18 years	HIV/Arthritis/Hypertension	Yes	Friend
PU007	Female	48	Unemployed	14 years	Hypertension	No	Husband
PU008	Male	56	Unemployed	19 years	Hypertension	Yes	Friend
PU009	Male	46	Employed	24 years	Diabetes/Hypertension/Depression	No	Wife
PU010	Male	57	Unemployed	19 years	Hypertension	Yes	Partner
PU011	Male	47	Self-employed	5 years	Hypertension	Yes	Partner
PU012	Female	57	Self-employed	18 years	Hypertension	No	None
PU013	Female	65	Retired	17 years	Hypertension/Diabetes/ liver failure	Yes	Partner
PU014	Male	72	Retired	7 years	Hypertension	No	None
PU015	Male	59	Unemployed	5 years	Hypertension/Diabetes	No	None
PU016	Male	46	Unemployed	26 years	Hypertension/Stroke	No	Brother and sister

**Table 3:** Participant characteristics Bulungula, Eastern Cape.

Study ID	Sex	Age	Employment	Time HIV+	Co-morbidity	Carer present	Main type of support
PR001	Female	61	Unemployed	9 years	Diabetes	Yes	Daughter
PR002	Female	59	Unemployed	18 years	Epilepsy	Yes	Daughter-in-law
PR003	Female	72	Retired	15 years	Hypertension	No	None
PR004	Female	60	EPWP	6 years	Heart condition	No	Daughter
PR005	Male	41	Farmer	8 years	Heart disease	No	Wife
PR006	Female	42	EPWP	6 years	Hypertension	Yes	Daughter
PR008	Female	40	Unemployed	9 years	Hypertension	No	Mother
PR009	Female	42	Unemployed	6 years	Hypertension	Yes	Sister-in-law
PR010	Female	63	Unemployed	15 years	Hypertension	No	None
PR011	Female	30	Unemployed	7 years	Hypertension	No	Mother
PR012	Female	48	Unemployed	7 years	Hypertension	No	Sister
PR013	Female	50	Unemployed	11 years	Hypertension	No	Sister
PR014	Male	34	Unemployed	6 years	Hypertension/Stomach condition	Yes	Mother
PR015	Female	63	Unemployed	long time	Hypertension/Cancer	Yes	Daughter

### The multiple facets of treatment burden for people living with multimorbidity

Having two or more chronic conditions affected the everyday life of all participants, but the impact varied across field-sites, between illnesses and the severity of conditions. All respondents had to take antiretroviral therapy (ART) daily, preferably after taking food. Depending on the type of ART regimen, commonly reported side effects included stomach cramps, fatigue, and headaches. However, participants who had been on the same ART regimen for more than 5 years reported minimal side-effects. Aside from taking ART, participants also had to monitor and take medications for their other conditions, including pills for hypertension or insulin injections or metformin and some had to monitor their blood sugars to help manage their diabetes. Participants suffering from hypertension complained about fatigue, headaches, having low energy and feelings of anxiety, which impacted their functional performance. Respondents with diabetes felt the most burdened, as self-managing diabetes required dietary and lifestyle changes, including strictly monitoring their sugar intake and required taking food with medication. Respondents previously suffering from TB shared the challenges of taking multiple drug therapy, which had many side-effects.

Monthly clinic visits were scheduled for HIV consultations and blood tests were taken twice a year to monitor viral load. Policies recommend that blood pressure is measured during HIV check-ups, but a few participants commented that this is not always done. On the structure of services, participants commented that apart from hypertension check-ups, HIV care is still largely separated from NCD care and people living with diabetes, epilepsy and other heart conditions had to schedule multiple appointments a month to ensure all their different chronic conditions were being managed appropriately. One participant also attended a separate clinic for her depression check-ups. Respondents had to combine these visits with paid work, housework, farming, and childcare responsibilities. Although the burden for participants with three or more conditions was high, they tried their best to stay adherent, attend clinic appointments and stay positive about their treatment burden.

“The best I can do is to adhere to the guidance from the nurses and not miss my clinic dates and take my medication duly. I have no stress about my conditions because I have accepted them.” PU008 [M56]

### Precariousness of income

A shortage of money, or losing income, influenced patient’s capacity to self-manage their conditions. This lack of material resources limited their functional performance, but the extent of the impact varied between urban and rural areas, between younger and older patients and between men and women. Most patients over the age of 60 year relied on the income of an old-age grant, which is a maximum of R1890 per month (~92 GBP per month). Households would pool grant money and additional available income to buy groceries, out-of-pocket medication, pay for transport to the clinic or to cover costs for health emergencies.

“The component of money is very difficult for me. We only have money at the end of the month [through a child support grant] for a short period of time. So, we take each day as it comes and try to not stress too much. Sometimes if I sell my mealies, I will have some bit of money, but it is not enough.” PR05 [M40]

Between the urban and rural field-sites, there was a significant difference in expenditure for clinic visits and health emergencies. In Bulungula, participants mostly walked to the clinic and would spend R10 (0.50 GBP) to cross the river with the ferry. Although time-consuming due to the large distances, costs were manageable when budgeted for, but when a health emergency would arise, rural respondents would have to fork out a minimum of R500 (25 GBP) to hire a private vehicle to get to the hospital, almost one third of their monthly income. To make it through the rest of the month, they would borrow money from family or neighbours to increase their resources or rely on food donations for their daily meals. Being indebted to others weighed heavily on patients and they felt constantly guilty about incurring expenses for treatment and having to ask others for help.

“I would walk to the road in extreme pain and get into the car which was R500. I do not even want to know the amount of debt my parents are in; I do not know how they will ever repay these debts they have incurred because of me and my illness.” PR14 [M34]

Participants in Bulungula who did not receive a disability or old-age grant shared the family’s child support grants for children or grandchildren (R460 per month, 23GBP) to cover household expenses. In this way, they were able to mobilise relational networks and increase their social capital. One advantage rural respondents had over urban participants was access to land for subsistence farming. This enabled them to grow their own crops and keep some livestock. However, as crops grow seasonally, participants complained about going hungry in August at the end of a long winter or when affected by drought. Although the land and produce were theirs, one participant shared that she struggled to produce her own crops, as she did not have the money to buy the seeds. Older participants complained that subsistence farming was strenuous on their ageing and ill bodies.

For participants in urban areas, grant support was also an important source of income, but some participants had formal employment or were self-employed. In general, urban areas in South Africa are better serviced and there are more job opportunities, but the demand on limited financial resources also differs, as there is rent to be paid and growing one own’s food is more difficult. Several participants lost their income due to deteriorating health or were laid off during the COVID-19 pandemic, which impacted the availability of their economic resources. This was especially challenging for participants aged between 50 and 60 years, as they were too young to apply for an old-age grant and did not qualify for a disability grant, although they felt too old to start a new job. This left them in an extremely precarious position, especially when other family members were unemployed, or had passed away.

“Since my husband passed away, there is no one who assists me. My youngest child is 20 and still at school. She needs help financially. So, I am alone, and I need financial help.” PU012 [F57]

Economic insecurity and a shortage of exploitable resources required participants in both areas to plan their finances carefully and make difficult trade-offs when making spending decisions. The need for school clothes, shoes or unexpected costs for funerals would strain their already tight family budgets, forcing them to make compromises, including delaying care or walking to the clinic instead of taking public transport. Participants also reserved funds for out-of-pocket medication which were not dispensed by the public health services, including ointments for cellulitis or treatment for gout. Sometimes, pain tablets would not last the whole month and had to be substituted with aspirin or borrowed medication from others:

“Right now, I am out of pills. I have asked my friend for pain pills. Mine ran out last week, I will only get more in April.” PU002 [F62].

During the COVID-19 pandemic, urban patients also purchased immune boosters and vitamin C tablets to boost their immune systems, as recommended by government guidelines. These immune boosters were costly and not dispensed at government clinics.

“I am paying for the boosters, vitamins, and stuff. To boost my body. At the pharmacy, they sell it for more than 30 rand, so I buy one at the time.” PU007 [F48]

When experiencing income insecurity, every financial decision had to be carefully weighed, which meant that participants could not always prioritise their own health. Several patients admitted being hungry regularly, skipping meals or having to take ART on an empty stomach, which is not recommended. People with diabetes, who were urged to eat fresh, healthy, and non-fatty foods, struggled to adhere to the recommended diet, as fresh fruit and vegetables were unaffordable or would not be available all year around. A few patients took up extra work or rented out rooms to raise money to buy the recommended food.

“I am getting an old age grant now. But I cannot say I am okay (financially), so I am selling sheep heads to add more, because I want to eat veggies like broccoli and spinach, the fresh foods.” PU005 [F63]

Participants in Bulungula had little opportunity to make additional, cash income. They relied on occasional work through the Expanded Public Works Programme (EPWP) which is unskilled and low paid work, in addition to subsistence farming. This compelled them to ask family members or neighbours for food or money, reciprocating when they were able to. This was accepted as custom and understood as part of Ubuntu – an African centred form of social support which stresses the importance of being connected to one another through kinship and community (68). Respondents spoke of how after a long day travelling to and from the clinic, they could ask neighbours to share their evening meal, how harvested maize and other vegetables were shared amongst households and how members of the community often lent each other money for basic supplies. Some participants spoke about receiving remittances from family members who worked in the city. However, despite the goodwill of others, some families struggled to put food on the table, which impacted their health. One participant was annoyed by healthcare workers’ apparent insensitivity to this situation:

“I need more nutritious food, but we cannot always afford this kind of food. It is tough as I told you. So, the doctors think I am not committed to improving my health but what do I do if there is no money? In fact, this whole thing about eating healthy begins to irritate me because they act like they cannot see this rural area, there is nothing. This food they speak off, where are we supposed to get it?” PR014 [M34]

Through the social support structure of Ubuntu, rural participants were able to maintain relational networks, often through kinship ties, and get additional practical help, as they received money or food from neighbours.

In Gugulethu, participants who did not have relational networks to rely on, experienced extreme precariousness when losing employment but being ineligible for a grant. One male respondent, who lived alone struggled to put food on the table, which resulted in severe weight loss.

“It is very hard, living without any work or a grant. I actually looked much better (before), this is not my face, this a face of hunger.” PU015 [M59]

His lack of support structure resulted in a lack of exploitable resources, which impacted his social capital and capacity to self-manage his conditions.

### Precarity of home and terrain

Living arrangements between urban and rural areas vary drastically. In rural Eastern Cape, traditional houses, or rondavels, are spread out over large areas of land. In urban Cape Town, people live in crowded settlements which are constantly expanding, due to ongoing urbanisation. Several urban participants lived in temporary, informal houses made of wood and corrugated iron, commonly known as shacks. These dwellings do not have sanitation or running water and people are compelled to share chemical toilets and communal taps with many others. This complicated their capacity to self-manage their conditions and compromised their overall health. Older and less mobile participants said they struggled to fetch water from outside taps or use outside toilets because of the uneven ground, the absence of pavements and poor lighting. One respondent, who had trouble walking due to complications of TB in her hip, commented:

“I am staying in a hokkie (shack). I was using the wheelchair for some time, but that became too hard for me (moving about in the shack). If only I could get my own house. It is also not safe to stay here.” PU005 [F63]

Other participants shared they were stressed as they did not have a permanent home and risked being evicted at any time by landlords from whom they rented temporary accommodation. For them, not having a permanent or formal address made it difficult to register for social services or receive deliveries, for example to get their medication delivered during lockdown. They were also located further from clinics, which increased their travelling times to the clinic, as well as the cost.

Rural participants did not experience such insecurity of tenure, as typically, the land they lived on had been settled by their families for several generations. However, many younger respondents were compelled to move to urban areas to seek education or jobs. One respondent spoke of how, when she was diagnosed with HIV and high blood pressure, she lost her job and had to move from the city to Bulungula to be closer to her family for support. She now lived in her sister’s house, who worked in the city and assisted her and her daughter financially. Another participant shared that he was forced to relocate to Bulungula from urban Mthatha when he lost his job in 2015 after being shot. His loss of income meant that the whole family had to relocate to the village, where they did some farming and lived on the child support grant (CSG) from his sister.

“We had an okay life, but all of that ended when I was shot. So, it has been hard having to adjust to a new way of life, I like my life here, my family is here but I wish we did not struggle. Everything here is very hard.” PR14 [M34]

He described how his relocation had a severe impact on the workload involved in managing his HIV and hypertension. He had to walk very far to the nearest clinic, could not afford a varied diet and had to borrow money from neighbours to get transport to go to an urban clinic to get treatment for chronic stomach pains.

All participants in remote Bulungula experienced challenges due to the distance of clinics, lack of transport and unforgiving terrain, which constrained their agency and increased their treatment burden. All except two had to travel a full day to the clinic by foot, which required advance logistical and financial planning. They described how the journey to the clinic involved taking a ferry across the river mouth, walking through thick forest or along a beach and navigating steep hills on difficult paths. To make their appointments, they had to carry cash for the ferry, to buy food along the way or to find a place to spend the night half-way through their journey to make it more manageable.

“I always try to ensure that I collect wood to sell so I can go to the clinic. Or if I cannot find anyone to sell to, I borrow money, even if I do not know how I am going to return it. I need R100 to go and come back, even if I do not eat, it’s fine.” PR06 [F46]

Participants in Bulungula also lacked basic amenities, such as running water within the home, and a lack of access to social services. Some patients had cell phones, but reception was limited and recharging difficult due to the lack of electricity. Data and airtime were also costly. Most rural respondents did not have access to mass media through TV or the internet. Consequently, respondents were relatively uninformed about their conditions and relied almost solely on the health information provided by clinic staff and community health workers (CHWs) from the Bulungula Incubator who visited patients at home.

“The nurses tell us about what we should do and what we should not do, but we do not receive any papers with information to take home. Also, the Community Health Workers come and check on me and see how I am doing and share information with me.” PR01[F61]

In addition, in rural areas low educational and literacy levels limited their ability to read pamphlets and posters at the clinics. This was very different in the urban areas, where participants had access to different media and had a higher level of literacy which enabled them to become more informed about their conditions.

### Precarity and the threat of violence

A major concern, especially for female respondents, was their fear of being a victim of violence when travelling to the clinic, further impacting access to health services. Several female participants in Gugulethu said they feared being assaulted enroute to the clinic or being robbed of their medication when returning from the pharmacy at the clinic. To get ahead of the queue for the clinic, they had to travel very early in the morning but felt vulnerable when waiting outside the gates before the clinic opened, fearing being robbed by totsi’s (thieves). During COVID-19, the situation worsened, as security officers at the clinic would only allow small groups of patients to enter the premises at a time, resulting in large crowds of patients gathering outside on the street with little security.

Participants from Bulungula complained that long distances and the limited available routes to the clinic posed risks for them and described how they tried to avoid travelling alone, as they were afraid of getting injured or assaulted when journeying across mountains or through forests. Sometimes, female respondents would wait for others to head out in the same direction, as they felt vulnerable walking on their own.

“When you are ready to go home from the clinic, you need to look for someone who you can walk with. But if they take too long, you will just take the chance and go into the forest alone. That is a huge risk that all patients are faced with.” PR012 [F48]

Not only did violence impact patients’ access to health services, but gender-based violence (GBV) and substance abuse were mentioned in households in both settings. This wrecked participants’ lives and fractured their relational networks. Tik (crystal methamphetamine) and alcohol addiction in families led to financial difficulties, intimate partner violence, mistrust, and stress. Some participants described how they felt unsafe living with abusive partners or family members, were afraid to leave their belongings when leaving the house and were not able to rely on their family members for help when experiencing a health emergency. One female was convinced that hearing that her son, only a teenager at the time, was on drugs, gave her a stroke.

“It happened because my son, he was using drugs. I was so stressed. After that, I had a stroke.” PU002 [F62]

Even though the patient recovered physically from the stroke, she still battled with anxiety and depression and was isolated from her social support system as she was afraid to leave the house, relying solely on care from her daughter. Similarly, when speaking about family relationships, another female respondent mentioned that her son, her main carer, overdosed and died at the age of 30. She mourned his death but was also relieved.

“My son used to help me, but he was a drug user. Tik. It was terrible. When he was high, he would go crazy. He was being aggressive, hissing at you, biting you. He would steal my things, but then the next day he is helping me. But since he died, nobody is stealing my things now.” PU005 [F63]

In the Eastern Cape, two female participants revealed that alcohol abuse by partners had negatively affected their marriages and financial circumstances. A large part of their income would be spent on beer and whiskey and when intoxicated, their partners would pick fights, physically and verbally abuse them, or leave the house for several days. One respondent cried when speaking about her marriage, as her husband fled home after stabbing his brother during a drunken brawl. She was mourning her brother-in-law’s death, whilst trying to provide for her family on her own and keep her children in school. She was very distressed and worried about her future, but was grateful for the support of her sister-in-law, who made sure she kept adherent to her HIV and hypertension medication.

In Cape Town, three male respondents spoke about their own history with alcohol and drug use, which made them irresponsible and, in some cases, led to HIV infection. Two of them stopped drinking all together, as it interfered with their medication regimes and overall health. Beating their bad habits, these men now supported others to embrace a healthy lifestyle, increasing their capacity.

“I had a neighbour; he was an alcoholic. He got this HIV while he was drunk. I changed that guy, he is right now. He quit alcohol, he is taking the ARVs now, so I made a difference in someone else’s life.” PU016[M46]

As recovered alcoholics, these participants gained a new sense of belonging through supporting others and in doing so, building new relational networks.

### Precarity and insufficient health service support

Participants’ capacity to manage the workload to live with HIV and other chronic conditions was affected by the type and level of support they received from their own relational networks and the structures provided by the state. Five urban participants complained that it was difficult to rely on the health services because of frequent stock outs of medication, which interrupted their self-management routines. The unavailability of monitoring tools was also reported, including blood pressure monitors and glucometers, and pillboxes to organise medication, which compromised their ability to control their conditions. Participants had to either buy their own supplies out of pocket or had to travel to the clinic to measure their blood pressure or glucose levels, which is time consuming and expensive. A patient with diabetes, who also had a partner with diabetes, said:

“It would be very helpful to have a glucose meter at home, because we will be able to see, and read what our sugar levels are.” PU013 [F65]

Information on their conditions was also not readily provided by the state health services. Participants felt that HIV information was more easily available, as they received counselling after their HIV diagnosis. However, health education and counselling after diagnosis of other chronic conditions, was scarce.

“I receive a lot of pamphlets and information about HIV, but I do not have much about my other condition (mental health). I would like to know more about how to manage my other conditions.” PU002 [F62]

Participants in rural Bulungula relied mostly on the health knowledge shared by the CHWs, who would inform them during home visits. The “nomakhayas” (CHWs in Isixhosa) visit patients at their homes to provide emotional support, share health information and remind them about their appointments. During the COVID-19 pandemic, they distributed masks and hand sanitizers and later coordinated the vaccination campaign. With health services far away and a lack of state support, the HCWs from the BI are a part of rural participants relational networks, as they assist with sense-making and self-monitoring their conditions. Participants greatly appreciated them:

“I can talk to my nomakhaya. I tell her about the challenges I am facing, and she helps me make decisions and gives me advice. She also follows up to check I am not defaulting on treatment and keeping to all my clinic appointments.” P06 [F42]

Compared to the continuous support offered by the CHWs in Bulungula, respondents in urban areas only occasionally spoke to a counsellor or social worker. Previously, HIV adherence clubs and peer-to-peer support groups, organised by local NGOs, were a part of urban participants’ support networks, but these were discontinued during the COVID-19 pandemic. Few participants had personal relationships with health staff, partly due to short consultation times and high staff turnover. In contrast to the rural respondents, many of the urban patients expressed frustration with the staff at the clinic:

“Every day they change things, they do not have the courtesy to tell you about what that change means, and when you ask – they shout at you sit here, go there and two hours passes, and you have not been helped and that is frustrating.” PU014 [M72]

Although some participants expressed their discontent with staff attitudes, most frustration was caused by organisational mismanagement at the urban clinic, including a stock-out of medication, understaffing and high staff turnover, which made it difficult for patients to build relationships with health staff. Lost folders were another annoyance:

“The clinic itself is fine, but the administration is horrible, they once lost my file for two months. I went without treatment for two months because they lost my folder.” PU010 [M57]

For urban patients, the longstanding organisational issues in clinics were exacerbated during the COVID-19 pandemic, as clinics closed during the various lockdowns and patients did not always have access to their treatment, as medication was not delivered to everyone in need. This adversely affected patients’ control over the health services and the capacity to adequately manage their multiple conditions, sometimes leading to serious health consequences and non-adherence to treatment.

## Discussion

These findings illustrate the accumulative impact that precariousness has on the lives and treatment burden of people living with multimorbidity in urban and rural low-income settings in South Africa. Our analysis identified various dimensions of precariousness which negatively impacted on patients’ capacity to self-manage multiple chronic conditions: these included economic insecurity, the threat of violence; difficult geographical terrain; insecure housing; and insufficient health service support.

Our findings echo results from other studies examining the impact of precariousness on patients in LMICs. These studies have similarly reported that limited financial resources, lack of insurance and financial instability are major barriers to self-management (69). Participants in other rural low-income settings have also reported experiencing a lack of health care providers and having to travel long distances to the clinic, commuting over rough terrain with poor road infrastructure whilst being at risk of violence (1, 69, 70).

This paper further illustrates that patient capacity to self-manage multimorbidity is complicated by the challenging biosocial complex in which people find themselves, as their health conditions have to be managed under conditions of health inequalities that are caused and sustained by a broad set of political, economic and social factors (28, 71). One patient summed this up by saying: “*the problem is not the treatment at the clinic, or staff, but the fact that the social conditions in the townships are bad. There is pollution, bad housing and hunger [PU009].”* Here, he highlights the structural inequalities experienced by South African patients, which even 25 years post-apartheid, are still prominent. Multiple chronic illnesses impacted every aspect of peoples’ lives, as they constantly needed to consider the financial cost of care-seeking, the social cost of caring for family members and the emotional costs of negotiating with health providers and support networks (22, 72, 73).

Living in constant precariousness made it nearly impossible for patients to be structurally resilient – as the loss of a job, closure or relocation of the clinic, or death of a family member could push them “over the edge,” as there is no financial buffer that can sustain them in times of need (74). Although most participants relied on state support through social grants, these barely covered basic food supplies, and often had to be shared within households. Especially in urban areas, this left participants between 50 and 60 years of age in extremely precarious positions, as they were considered “too healthy” to be on a disability grant and “too young” to qualify for an old-age grant. Disability grants are only paid to chronically ill patients who are unstable and sick; once they are on treatment and stable, they lose their grant, as they are considered “fit to work.” Many participants considered this grant a “lifeline” and experienced stress and anxiety when it was withdrawn. Rural participants often relied on financial handouts from family, friends, and neighbours. This support was offered during health crises, but long-term, sustained financial help was rarely available, especially when participants were not able to reciprocate or pay back their debt.

Precariousness can be accumulative and cyclical in nature, as financial instability can lead to problems accessing health services, securing food and safe housing, and magnify tensions in relationships. This accumulation of precarity was amplified during the COVID-19 pandemic with its strictly imposed lockdowns when people experienced increased levels of economic uncertainty, disruption of health services; increased crime and intensified tension in relationships (75). Manderson and Warren (70) describe the cyclical nature of precariousness as “recursive cascades,” encapsulating the impact that growing impoverishment has on increasing ill-health. Describing a three-tiered system, the recursive cascade explains how social and relational factors plus economic and practical resources worsen chronic conditions (70).

**Figure 1:**
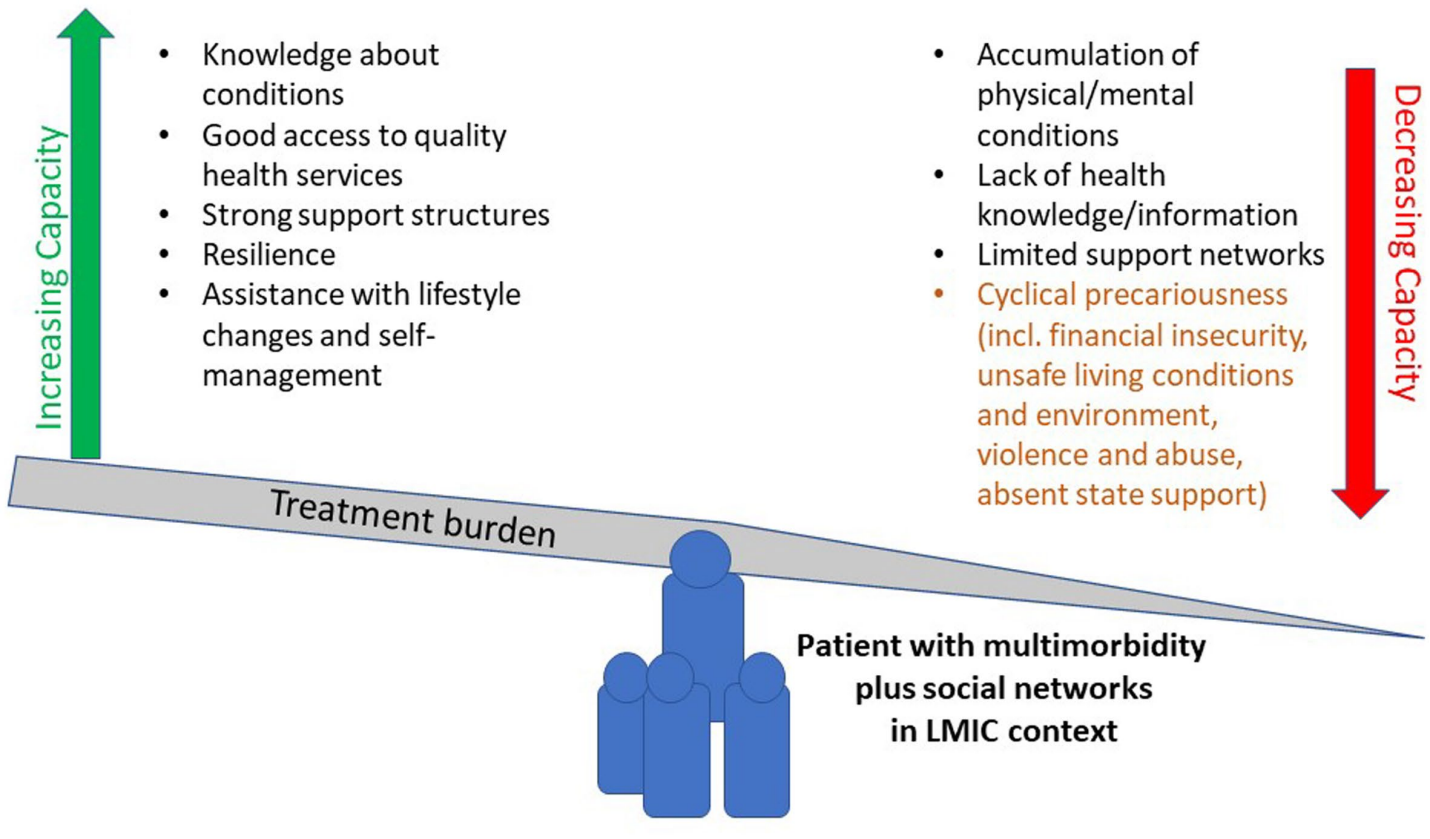
BoTT with the integration of cyclical precariousness.

Whilst BoTT does include the need to mobilise material resources to navigate treatment burden (*exploitable resources*) and acknowledges how the lack of both informational and material resources (*social capital*) can put patients self-management and support structures under threat, findings in this paper describe that cyclical precariousness influences every aspect of patients’ lives and ability to self-manage their health. Cyclical precariousness, in this LMIC context, is not just the inability to exploit opportunities to access health care resources but sums up the difficulties of managing long-term conditions when experiencing constant financial insecurities, which impact on patients’ basic needs such as shelter, safety, food security and social support. Furthermore, the current BoTT does not sufficiently emphasise the impact that a lack of financial resources has on patients’ ability to mobilise social support which in turn, diminishes social capital and self-management capacity. We would therefore suggest that the BoTT, which was developed in a high-income setting, needs to be adapted to include “cyclical precariousness” to make it a more applicable and useful theoretical model for use in LMIC contexts. Our findings suggest that cyclical precariousness negatively impacts patients’ capacity to self-manage their conditions and in turn, increases their treatment burden, resulting in negative health outcomes (see Figure 1).

Although the precise terms are not used, the phenomena of cyclical precariousness and recursive cascades are also clearly reported in other treatment burden studies in LMICs, including amongst patients with chronic conditions in Ghana, Argentina and Malawi. These patients reported that due to financial constraints, patients struggled to cope with their treatment burden and keep up with the systems demands, due to a lack availability of health services (8, 42, 43). It is also important to recognise that chronic patients from vulnerable backgrounds are often compelled to prioritise their economic survival over controlling their health conditions (69). Underprivileged groups in HICs may similarly experience the impact of financial instability: For example, undocumented migrants in the US, who often rely solely on emergency care, are cut off from relational networks and have no access to government support (76, 77). After the financial crisis in 2008, many European countries cut welfare support, pushing vulnerable groups into precariousness, leading to homelessness, reliance on foodbanks and poorer health outcomes (1, 78, 79).

Cyclical precariousness also resulted in challenges for patients when navigating care in a siloed, disease focused, and overburdened health system, which perpetuates structural vulnerabilities, increases out-of-pocket costs and forces patients to be more reliant on their support networks (12, 80). As South Africa is attempting to move towards a patient-centred integrated care system, it is essential to consider the impact of precariousness on patients’ capacity to self-manage chronic illnesses. As it stands, the ICDM model expects patients to take on more responsibility to self-manage their conditions, but does not consider the additional financial and social costs involved nor the increasing self-care demands of coping with multimorbidity compared to a single disease (36). In our study, it was clear that rural participants relied heavily on the support and guidance of HCWs. This increased their self-management capacity even when living in precarious conditions, as the CHWs informed them about their conditions, assisted with their treatment and offered emotional support. This suggests that CHWs could play an invaluable role in reengineering the primary healthcare system to improve chronic care particularly in the context of a growing population living with multimorbidity in these settings. Lastly, when informing patients about treatment for multimorbidity, communication channels and language should be carefully considered, as this study showed that literacy levels and access to media varied considerably between urban and rural participants.

### Strengths and limitations

Our study has some limitations. Firstly, we intended to recruit an equal sample of men and women in the urban and rural areas, but only succeeded in including 2 men in the rural sample. This was due to the migration of men to urban areas for work, and their reduced engagement in HIV/NCD care compared to women (81, 82). Secondly, we only recruited two participants with self-reported depression, as mental illnesses are underreported in this context, and therefore our work cannot provide insights into additional issues experienced by people with mixed mental-and physical multimorbidity (83). Thirdly, all respondents lived with HIV/NCD multimorbidity, which means that we did not gain insights about the experience of patients with multimorbidity that did not include HIV as one of the combination of conditions, so additional work with patients with other patterns of multimorbidity would be worth exploring in future research. A key strength is the comparative nature of the study, as urban and rural interviews revealed a great variation within a common experience, which demonstrates its potential transferability or relevance to a wider or alternative setting. Participants accounts of living with multimorbidity differed, based on their culture, background, and social meanings. A further strength is the prolonged engagement within the study sites, as we regularly visited the clinic in Gugulethu and stayed in the local community of Bulungula for 2 weeks. Therefore, the data collected provided rich insights on peoples’ treatment burden and we were able to reach data saturation in both settings as previously reported (46). The use of well-established theories of treatment burden to frame the research and analysis, enhances the potential for replication of this study in other settings (84).

## Conclusion

Whilst we recognise that this is a small study and therefore findings cannot be generalised for the population, we believe that this study contributes meaningfully to the small body of literature that explores and advances our understanding of the personal, lived experiences of people with multimorbidity in low-income Sub-Saharan settings. Our work demonstrates that the CuCoM and the BoTT are viable theoretical underpinnings to investigate treatment burden in low-income settings. However, our findings also emphasise the impact of cyclical precariousness on patient workload and capacity, and we suggest that this aspect should be incorporated as an additional dimension to the BoTT. This could make the framework and future treatment burden measures more appropriate for use in South Africa. Our work also showed that support structures, especially in rural settings, positively impacted patients’ self-management capacity, even when living in precariousness. To respond to the increased multimorbidity health needs in low-income settings in South Africa, the results point to the need to introduce a basic income grant that improves the standard of living, which can enhance self-management capacity and health outcomes. Actively including family and community support networks in self-management interventions can further increase capacity. To achieve person-centred care, support must be tailored to address the varying capacity of people to self-manage their multiple chronic illnesses.

## Data availability statement

The data set used and analysed during this study is available from the corresponding author on reasonable request. Please contact Dr van Pinxteren, Myrna.vanpinxteren@uct.ac.za.

## Ethics statement

The studies involving human participants were reviewed and approved by University of Cape Town Human Research Ethics Committee. The patients/participants provided their written informed consent to participate in this study.

## Author contributions

MP data collection and analysis, conceptualisation and write up of the paper. NM data collection and analysis, extensive intellectual input in the paper. KM conceptualisation of research project, extensive editing of the paper. FM conceptualisation of research project, extensive intellectual input, and editing. CM conceptualisation of research project, extensive intellectual input, and editing. NL conceptualisation of research project, extensive intellectual input, and editing. All authors contributed to the article and approved the submitted version.

## Funding

This work was supported by the United Kingdom Medical Research Council [Grant number MR/T03775X/1]. CM is partly supported by the NIHR North Thames Applied Research Collaborative [grant number NIHR200163].

## Conflict of interest

The authors declare that the research was conducted in the absence of any commercial or financial relationships that could be construed as a potential conflict of interest.

## Publisher’s note

All claims expressed in this article are solely those of the authors and do not necessarily represent those of their affiliated organizations, or those of the publisher, the editors and the reviewers. Any product that may be evaluated in this article, or claim that may be made by its manufacturer, is not guaranteed or endorsed by the publisher.

## References

[1] McKee M Reeves A Clair A Stuckler D. Living on the edge: precariousness and why it matters for health. Arch Public Health. (2017) 75:1–10. doi: 10.1186/s13690-017-0183-y 28270912 PMC5335798

[2] Farmer P. An anthropology of structural violence. Curr Anthropol. (2004) 45:305–25. doi: 10.1086/382250

[3] Butler J. Performativity, Precarity and sexual politics. AIBR Revista de Antropología Iberoamericana. (2009) 4

[4] Butler J. Precarious life: The powers of mourning and violence: Verso; (2004).

[5] Mills E. “When the skies fight”: HIV, violence and pathways of precarity in South Africa. Reprod Health Matters. (2016) 24:85–95. doi: 10.1016/j.rhm.2016.04.006, PMID: 27578342

[6] Mendenhall E Norris SA. When HIV is ordinary and diabetes new: remaking suffering in a south African township. Glob Public Health. (2015) 10:449–62. doi: 10.1080/17441692.2014.998698, PMID: 25643001 PMC4353257

[7] Fast D Bukusi D Moyer E. The knife's edge: masculinities and precarity in East Africa. Soc Sci Med. (2020) 258:113097. doi: 10.1016/j.socscimed.2020.113097, PMID: 32540514

[8] Roberti J Alonso JP Blas L May C. How do social and economic vulnerabilities shape the work of participating in care? Everyday experiences of people living with kidney failure in Argentina. Soc Sci Med. (2022) 293:114666. doi: 10.1016/j.socscimed.2021.114666, PMID: 34952327

[9] Chitiga M Owusu-Sekyere E Tsoanamatsie N. Income inequality and limitations of the Gini index: The case of South Africa. (2014).

[10] Mayosi BM Benatar SR. Health and health Care in South Africa — 20 years after Mandela. N Engl J Med. (2014) 371:1344–53. doi: 10.1056/NEJMsr1405012, PMID: 25265493

[11] Mayosi BM Lawn JE Van Niekerk A Bradshaw D Karim SSA Coovadia HM. Health in South Africa: changes and challenges since 2009. Lancet. (2012) 380:2029–43. doi: 10.1016/S0140-6736(12)61814-5, PMID: 23201214

[12] Coovadia H Jewkes R Barron P Sanders D McIntyre D. The health and health system of South Africa: historical roots of current public health challenges. Lancet. (2009) 374:817–34. doi: 10.1016/S0140-6736(09)60951-X, PMID: 19709728

[13] Gumede V. Revisiting poverty, human development and inequality in democratic South Africa. Indian J Hum Dev. (2021) 15:183–99. doi: 10.1177/09737030211032961

[14] Eye Witness News. Social grants second most important source of income in SA-STATS SA (2019).

[15] Stats SA. The extent of food security in South Africa (2017). Available at: https://www.statssa.gov.za/?p=12135

[16] Stats SA. General household survey. 2016.

[17] Scott V Schaay N Schneider H Sanders D. Addressing social determinants of health in South Africa: the journey continues. South African Health Rev. (2017) 2017:77–87.

[18] Oni T McGrath N BeLue R Roderick P Colagiuri S May CR. Chronic diseases and multi-morbidity-a conceptual modification to the WHO ICCC model for countries in health transition. BMC Public Health. (2014) 14:1–7. doi: 10.1186/1471-2458-14-575 24912531 PMC4071801

[19] Onoya D Mokhele I Sineke T Mngoma B Moolla A Vujovic M. Health provider perspectives on the implementation of the same-day-ART initiation policy in the Gauteng province of South Africa. Health Res Policy Syst. (2021) 19:1–12. doi: 10.1186/s12961-020-00673-y 33407574 PMC7789550

[20] Roomaney RA van Wyk B Turawa EB Pillay-van WV. Multimorbidity in South Africa: a systematic review of prevalence studies. BMJ Open. (2021) 11:e048676. doi: 10.1136/bmjopen-2021-048676, PMID: 34615675 PMC8496399

[21] Wong EB Olivier S Gunda R Koole O Surujdeen A Gareta D. Convergence of infectious and non-communicable disease epidemics in rural South Africa: a cross-sectional, population-based multimorbidity study. Lancet Glob Health. (2021) 9:e967–76. doi: 10.1016/S2214-109X(21)00176-5, PMID: 34143995 PMC8220132

[22] Bosire EN Mendenhall E Norris SA Goudge J. Patient-centred care for patients with diabetes and HIV at a public tertiary hospital in South Africa: an ethnographic study. Int J Health Policy Manag. (2021) 10:534–45. doi: 10.34172/ijhpm.2020.65, PMID: 32610758 PMC9278375

[23] Mendenhall E Kohrt BA Norris SA Ndetei D Prabhakaran D. Non-communicable disease syndemics: poverty, depression, and diabetes among low-income populations. Lancet. (2017) 389:951–63. doi: 10.1016/S0140-6736(17)30402-6, PMID: 28271846 PMC5491333

[24] George A. Neither passive nor perverse: government rural health assistants as social beings In: Sheikh K George A, editors. Health providers in India: On the frontlines of change. New Delhi: Routledge (2010)

[25] Mayosi BM Flisher AJ Lalloo UG Sitas F Tollman SM Bradshaw D. The burden of non-communicable diseases in South Africa. Lancet. (2009) 374:934–47. doi: 10.1016/S0140-6736(09)61087-4 19709736

[26] Lalkhen H Mash R. Multimorbidity in non-communicable diseases in south African primary healthcare. S Afr Med J. (2015) 105:134–8. doi: 10.7196/SAMJ.8696, PMID: 26242533

[27] Oni T Youngblood E Boulle A McGrath N Wilkinson RJ Levitt NS. Patterns of HIV, TB, and non-communicable disease multi-morbidity in peri-urban South Africa-a cross sectional study. BMC Infect Dis. (2015) 15:1–8. doi: 10.1186/s12879-015-0750-1 25595711 PMC4300166

[28] Roomaney RA Wyk BV Wyk VP. Decolonising multimorbidity? Research gaps in low and middle-income countries. Pan Afr Med J. (2022) 41:140. doi: 10.11604/pamj.2022.41.140.32104 35519173 PMC9034556

[29] Ngobeni V Breitenbach MC Aye GC. Technical efficiency of provincial public healthcare in South Africa. Cost Eff Resour Allocation. (2020) 18:3. doi: 10.1186/s12962-020-0199-y, PMID: 32002018 PMC6986147

[30] Matima R Murphy K Levitt NS BeLue R Oni T. A qualitative study on the experiences and perspectives of public sector patients in Cape Town in managing the workload of demands of HIV and type 2 diabetes multimorbidity. PLoS One. (2018) 13:e0194191. doi: 10.1371/journal.pone.0194191, PMID: 29538415 PMC5851623

[31] Levitt NS Steyn K Dave J Bradshaw D. Chronic noncommunicable diseases and HIV-AIDS on a collision course: relevance for health care delivery, particularly in low-resource settings—insights from South Africa. Am J Clin Nutr. (2011) 94:S1690–6. doi: 10.3945/ajcn.111.019075, PMID: 22089433 PMC3226022

[32] Grimsrud A Wilkinson L. Acceleration of differentiated service delivery for HIV treatment in sub-Saharan Africa during COVID-19. J Int AIDS Soc. (2021) 24:e25704. doi: 10.1002/jia2.25704, PMID: 34105884 PMC8188395

[33] Grimsrud A Lesosky M Kalombo C Bekker L-G Myer L. Community-based adherence clubs for the management of stable antiretroviral therapy patients in Cape Town, South Africa: a cohort study. J Acquir Immune Defic Syndr. (2016) 71:e16–23. doi: 10.1097/QAI.0000000000000863 26473798

[34] Burger R Christian C. Access to health care in post-apartheid South Africa: availability, affordability, acceptability. Health Econ Policy Law. (2020) 15:43–55. doi: 10.1017/S1744133118000300, PMID: 29996951

[35] Nachega JB Kapata N Sam-Agudu NA Decloedt EH Katoto PD Nagu T. Minimizing the impact of the triple burden of COVID-19, tuberculosis and HIV on health services in sub-Saharan Africa. Int J Infect Dis. (2021) 113:S16–21. doi: 10.1016/j.ijid.2021.03.038, PMID: 33757874 PMC7980520

[36] Mahomed OH Asmall S Freeman M. An integrated chronic disease management model: a diagonal approach to health system strengthening in South Africa. J Health Care Poor Underserved. (2014) 25:1723–9. doi: 10.1353/hpu.2014.0176, PMID: 25418238

[37] Ameh S Klipstein-Grobusch K D’ambruoso L Kahn K Tollman SM Gómez-Olivé FX. Quality of integrated chronic disease care in rural South Africa: user and provider perspectives. Health Policy Plan. (2017) 32:257–66. doi: 10.1093/heapol/czw118, PMID: 28207046 PMC5400067

[38] Ameh S. Evaluation of an integrated HIV and hypertension management model in rural South Africa: a mixed methods approach. Glob Health Action. (2020) 13:1750216. doi: 10.1080/16549716.2020.1750216, PMID: 32316885 PMC7191904

[39] Shippee ND Shah ND May CR Mair FS Montori VM. Cumulative complexity: a functional, patient-centered model of patient complexity can improve research and practice. J Clin Epidemiol. (2012) 65:1041–51. doi: 10.1016/j.jclinepi.2012.05.005, PMID: 22910536

[40] May CR Eton DT Boehmer K Gallacher K Hunt K Mac Donald S. Rethinking the patient: using burden of treatment theory to understand the changing dynamics of illness. BMC Health Serv Res. (2014) 14:1–11. doi: 10.1186/1472-6963-14-281 24969758 PMC4080515

[41] Boehmer KR Gionfriddo MR Rodriguez-Gutierrez R Leppin AL Hargraves I May CR. Patient capacity and constraints in the experience of chronic disease: a qualitative systematic review and thematic synthesis. BMC Fam Pract. (2016) 17:1–23. doi: 10.1186/s12875-016-0525-9 27585439 PMC5009523

[42] Chikumbu EF Bunn C Kasenda S Dube A Phiri-Makwakwa E Jani BD. Experiences of multimorbidity in urban and rural Malawi: an interview study of burdens of treatment and lack of treatment. PLOS Global Public Health. (2022) 2:e0000139. doi: 10.1371/journal.pgph.0000139 36962280 PMC10021162

[43] Morgan SA Eyles C Roderick PJ Adongo PB Hill AG. Women living with multi-morbidity in the Greater Accra region of Ghana: a qualitative study guided by the cumulative complexity model. J Biosoc Sci. (2019) 51:562–77. doi: 10.1017/S0021932018000342, PMID: 30472965

[44] Eton DT de Oliveira DR Egginton JS Ridgeway JL Odell L May CR. Building a measurement framework of burden of treatment in complex patients with chronic conditions: a qualitative study. Patient Relat Outcome Meas. (2012) 3:39. doi: 10.2147/PROM.S34681 23185121 PMC3506008

[45] Mair FS May CR. Thinking about the burden of treatment British Medical Journal Publishing Group (2014).

[46] Van Pinxteren M Mbokazi N Murphy K Mair FS May C Levitt NS. Using qualitative study designs to understand treatment burden and capacity for self-care among patients with HIV/NCD multimorbidity in South Africa: a methods paper. J Multimorb Comorb. Preprint 10.1177/26335565231168041 PMC10088413 37057034

[47] Owens J. Liberating voices through narrative methods: the case for an interpretive research approach. Disability Soc. (2007) 22:299–313. doi: 10.1080/09687590701259617

[48] Roest B Milota M Leget C. Developing new ways to listen: the value of narrative approaches in empirical (bio)ethics. BMC Med Ethics. (2021) 22:124. doi: 10.1186/s12910-021-00691-7, PMID: 34530832 PMC8447625

[49] Tran V-T Harrington M Montori VM Barnes C Wicks P Ravaud P. Adaptation and validation of the treatment burden questionnaire (TBQ) in English using an internet platform. BMC Med. (2014) 12:109. doi: 10.1186/1741-7015-12-109 24989988 PMC4098922

[50] Hunter C Chew-Graham C Langer S Stenhoff A Drinkwater J Guthrie E. A qualitative study of patient choices in using emergency health care for long-term conditions: the importance of candidacy and recursivity. Patient Educ Couns. (2013) 93:335–41. doi: 10.1016/j.pec.2013.06.001, PMID: 23906651

[51] Gallacher K Jani B Morrison D Macdonald S Blane D Erwin P. Qualitative systematic reviews of treatment burden in stroke, heart failure and diabetes-methodological challenges and solutions. BMC Med Res Methodol. (2013) 13:10. doi: 10.1186/1471-2288-13-10 23356353 PMC3568050

[52] Field S. Lost communities, living memories: remembering forced removals in Cape Town: New Africa Books. 2001.

[53] Poverty and Equality Initiative. Youth explorer (2023). Available at: https://www.youthexplorer.org.za/ (Accessed September 05, 2019).

[54] Porter G Phillips-Howard K. Agricultural issues in the former homelands of South Africa: the Transkei. Rev Afr Polit Econ. (1997) 24:185–202. doi: 10.1080/03056249708704252

[55] Baiyegunhi L Fraser GC. Determinants of household poverty dynamics in rural regions of the Eastern Cape Province, South Africa. (2010).

[56] Westaway MS Seager JR Rheeder P Van Zyl DG. The effects of social support on health, well-being and management of diabetes mellitus: a black south African perspective. Ethn Health. (2005) 10:73–89. doi: 10.1080/1355785052000323047, PMID: 15841588

[57] National Department of Health SSA, South African Medical Research Council. South Africa demographic and health survey 2016. Pretoria, South Africa and Rockville, Maryland, USA. (2019).

[58] Curley J Grinstein-Weiss M. A comparative analysis of rural and urban saving performance in individual development accounts. (2003).

[59] Campbell S Greenwood M Prior S Shearer T Walkem K Young S. Purposive sampling: complex or simple? Research case examples. J Res Nurs. (2020) 25:652–61. doi: 10.1177/1744987120927206, PMID: 34394687 PMC7932468

[60] Ulin PR Robinson ET Tolley EE. Qualitative methods in public health. San Francisco, CA: Jossey Bass (2005).

[61] University of Cape Town. Faculty of Health Sciences (FHS): Summary of approach to research in time of COVID-19 outbreak. (2022). Available at: http://www.health.uct.ac.za/sites/default/files/image_tool/images/116/UCT%20FHS%20Research%20in%20time%20of%20COVID-19%20outbreak_14%20Apr%202020.pdf. (Accessed July 20, 2022).

[62] Pope C Ziebland S Mays N. Analysing qualitative data. BMJ. (2000) 320:114–6. doi: 10.1136/bmj.320.7227.114, PMID: 10625273 PMC1117368

[63] Smith J Firth J. Qualitative data analysis: the framework approach. Nurse Res. (2011) 18:52–62. doi: 10.7748/nr2011.01.18.2.52.c8284 21319484

[64] Hackett A Strickland K. Using the framework approach to analyse qualitative data: a worked example. Nurse Res. (2018) 26:8–13. doi: 10.7748/nr.2018.e1580 30215482

[65] Ritchie J Spencer L O’Connor W. Carrying out qualitative analysis. In *Qualitative research practice: A guide for social science students and researchers*. (2003). 219–262.

[66] Parkinson S Eatough V Holmes J Stapley E Midgley N. Framework analysis: a worked example of a study exploring young people’s experiences of depression. Qual Res Psychol. (2016) 13:109–29. doi: 10.1080/14780887.2015.1119228

[67] World Medical Association. World medical association declaration of Helsinki: ethical principles for medical research involving human subjects. JAMA. (2013) 310:2191–4. doi: 10.1001/jama.2013.281053 24141714

[68] Mugumbate JR Chereni A. Now, the theory of Ubuntu has its space in social work. Afr J Soc Work. (2020) 10

[69] Schulman-Green D Jaser SS Park C Whittemore R. A metasynthesis of factors affecting self-management of chronic illness. J Adv Nurs. (2016) 72:1469–89. doi: 10.1111/jan.12902, PMID: 26781649 PMC4891247

[70] Manderson L Warren N. “Just one thing after another”: recursive cascades and chronic conditions. Med Anthropol Q. (2016) 30:479–97. doi: 10.1111/maq.12277, PMID: 26756733

[71] Singer M Bulled N Ostrach B Mendenhall E. Syndemics and the biosocial conception of health. Lancet. (2017) 389:941–50. doi: 10.1016/S0140-6736(17)30003-X, PMID: 28271845

[72] Chew-Graham C O’Toole L Taylor J Salisbury C. ‘Multimorbidity’: an acceptable term for patients or time for a rebrand? Br J Gen Pract. (2019) 69:372–3. doi: 10.3399/bjgp19X704681, PMID: 31345795 PMC6650114

[73] Bosire EN. Patients’ experiences of comorbid HIV/AIDS and diabetes care and management in Soweto, South Africa. Qual Health Res. (2021) 31:373–84. doi: 10.1177/1049732320967917, PMID: 33150848

[74] Náfrádi L Kostova Z Nakamoto K Schulz PJ. The doctor–patient relationship and patient resilience in chronic pain: a qualitative approach to patients’ perspectives. Chronic Illn. (2018) 14:256–70. doi: 10.1177/1742395317739961, PMID: 29096534

[75] Mbunge E. Effects of COVID-19 in south African health system and society: an explanatory study. Diabetes Metab Syndr Clin Res Rev. (2020) 14:1809–14. doi: 10.1016/j.dsx.2020.09.016, PMID: 32956925 PMC7485444

[76] Cervantes L Fischer S Berlinger N Zabalaga M Camacho C Linas S. The illness experience of undocumented immigrants with end-stage renal disease. JAMA Intern Med. (2017) 177:529–35. doi: 10.1001/jamainternmed.2016.8865, PMID: 28166331

[77] Wild V Dawson A. Migration: a core public health ethics issue. Public Health. (2018) 158:66–70. doi: 10.1016/j.puhe.2018.02.023, PMID: 29606282

[78] Lopez Bernal JA Gasparrini A Artundo CM McKee M. The effect of the late 2000s financial crisis on suicides in Spain: an interrupted time-series analysis. Eur J Public Health. (2013) 23:732–6. doi: 10.1093/eurpub/ckt083, PMID: 23804080

[79] Kentikelenis A Karanikolos M Reeves A McKee M Stuckler D. Greece's health crisis: from austerity to denialism. Lancet. (2014) 383:748–53. doi: 10.1016/S0140-6736(13)62291-6, PMID: 24560058

[80] Knight L Schatz E Mukumbang FC. “I attend at vanguard and I attend here as well”: barriers to accessing healthcare services among older south Africans with HIV and non-communicable diseases. Int J Equity Health. (2018) 17:1–10. doi: 10.1186/s12939-018-0863-4 30227859 PMC6145370

[81] Cornell M Johnson LF Wood R Tanser F Fox MP Prozesky H. Twelve-year mortality in adults initiating antiretroviral therapy in South Africa. J Int AIDS Soc. (2017) 20:21902. doi: 10.7448/IAS.20.1.21902, PMID: 28953328 PMC5640314

[82] Smith PJ Davey DJ Green H Cornell M Bekker L-G. Reaching underserved south Africans with integrated chronic disease screening and mobile HIV counselling and testing: a retrospective, longitudinal study conducted in Cape Town. PLoS One. (2021) 16:e0249600. doi: 10.1371/journal.pone.0249600, PMID: 33945540 PMC8096085

[83] Pillay Y. State of mental health and illness in South Africa. SAGE Publications Sage UK: London, England; 2019. 463–466.

[84] Morse JM. Critical analysis of strategies for determining rigor in qualitative inquiry. Qual Health Res. (2015) 25:1212–22. doi: 10.1177/1049732315588501, PMID: 26184336

